# Patient-Reported Symptoms and Sequelae 12 Months After COVID-19 in Hospitalized Adults: A Multicenter Long-Term Follow-Up Study

**DOI:** 10.3389/fmed.2022.834354

**Published:** 2022-03-22

**Authors:** Agnese Comelli, Giulia Viero, Greta Bettini, Alessandro Nobili, Mauro Tettamanti, Alessia Antonella Galbussera, Antonio Muscatello, Marco Mantero, Ciro Canetta, Filippo Martinelli Boneschi, Andrea Arighi, Paolo Brambilla, Maurizio Vecchi, Pietro Lampertico, Paolo Bonfanti, Marco Contoli, Francesco Blasi, Andrea Gori, Alessandra Bandera

**Affiliations:** ^1^Infectious Diseases Unit, Fondazione Istituto di Ricovero e Cura a Carattere Scientifico (IRCCS) Ca' Granda Ospedale Maggiore Policlinico, Milan, Italy; ^2^Department of Pathophysiology and Transplantation, University of Milan, Milan, Italy; ^3^Department of Health Policy, Istituto di Ricerche Farmacologiche Mario Negri Istituti di Ricovero e Cura a Carattere Scientifico (IRCCS), Milan, Italy; ^4^Respiratory Unit and Cystic Fibrosis Adult Centre, Internal Medicine Department, Fondazione Istituto di Ricovero e Cura a Carattere Scientifico (IRCCS) Ca' Granda Ospedale Maggiore Policlinico, Milan, Italy; ^5^Acute Medical Unit, Fondazione Istituto di Ricovero e Cura a Carattere Scientifico (IRCCS) Ca' Granda Ospedale Maggiore Policlinico, Milan, Italy; ^6^Neurology Unit, Fondazione Istituto di Ricovero e Cura a Carattere Scientifico (IRCCS) Ca' Granda Ospedale Maggiore Policlinico, Milan, Italy; ^7^Department of Neurosciences and Mental Health, Fondazione Istituto di Ricovero e Cura a Carattere Scientifico (IRCCS) Ca' Granda Ospedale Maggiore Policlinico, Milan, Italy; ^8^Gastroenterology and Endoscopy Unit, Fondazione Istituto di Ricovero e Cura a Carattere Scientifico (IRCCS) Ca' Granda Ospedale Maggiore Policlinico, Milan, Italy; ^9^CRC “A. M. and A. Migliavacca” Center for Liver Disease, Fondazione Istituto di Ricovero e Cura a Carattere Scientifico (IRCCS) Ca' Granda Ospedale Maggiore Policlinico, Milan, Italy; ^10^Infectious Diseases Unit, Azienda Socio Sanitaria Territoriale Monza, San Gerardo Hospital, Monza, Italy; ^11^Research Centre on Asthma and Chronic Obstructive Pulmonary Disease, Department of Medical Sciences, University of Ferrara, Ferrara, Italy

**Keywords:** long COVID-19, SARS-CoV-2, long-term sequelae, COVID-19, dyspnea

## Abstract

**Objective:**

Our knowledge on the long-term consequences of COVID-19 is still scarce despite the clinical relevance of persisting syndrome. The aim of this study was to analyze patient-reported outcomes, including assessment by specific questionnaires of health impairment and symptoms.

**Methods:**

This is a prospective, observational and multicenter cohort study coordinated by Fondazione IRCSS Ca' Granda Ospedale Maggiore Policlinico di Milano and Istituto di Ricerche Farmacologiche Mario Negri IRCCS including eight hospitals located in North and Central Italy. A telephone interview to assess rehospitalization, access to health care resources, general health status subjective evaluation, and symptoms was performed at 12 months after the discharge in patients admitted to hospital because of COVID-19 from February 2020 to the end of May 2020.

**Results:**

Among the 776 patients discharged alive, 44 (5.7%) died, 456 subjects (58.8%) completed the questionnaire and 276 (35.6%) were not reachable or refused to join the telephone interview. The mean age of the study population was 59.4 years (SD 14.1), 69.8% of individuals needed oxygen support during hospitalization and 10.4% were admitted to ICU. Overall, 91.7% of participants reported at least one symptom/sequela at 12 months. Exertional dyspnea (71.7%), fatigue (54.6%), and gastrointestinal symptoms (32.8%) were the most reported ones. Health issues after discharge including hospitalization or access to emergency room were described by 19.4% of subjects. Female and presence of comorbidities were independent predictors of whealth impairment and presence of ≥2 symptoms/sequelae after 12 months from hospitalization for COVID-19.

**Conclusions:**

Patient-reported symptoms and sequelae, principally dyspnea and fatigue, are found in most individuals even 12 months from COVID-19 hospitalization. Long-term follow-up based on patient-centered outcome can contribute to plan tailored interventions.

## Introduction

Since the first documented case of pneumonia related to SARS-CoV-2 in December 2019, progresses have been made on management of COVID-19.

However, researchers have focused mainly on the acute phase, attention has lately been shifting to post-COVID-19 signs and symptoms and, as a consequence, there is still very limited information on the sequalae of COVID-19 (1). The exact nature and prevalence of persistent symptoms after SARS-CoV-2 infection are not known, but an increasing number of studies are reporting a high incidence of sequelae during the convalescence months, defined as “*long-COVID-19*,” which recently obtained a formal definition by WHO (2).

Long-COVID-19 sequelae were reported by several studies and range from cough, dyspnea, fatigue, chest pain, migraine but also neurocognitive symptoms such as depression, anxiety, and insomnia (3, 4). During early COVID-19 convalescence, these clinical sequelae seem to be related to gender, age, BMI, and clinical severity during hospitalization (5, 6).

Unfortunately, robust data are lacking in terms of duration of this syndrome, risk factors, long term differences among patients with severe COVID-19 and individuals who experienced only mild or no symptoms, best methods to follow those patients and potential treatment.

With that in mind, it is likely that patients recovering from COVID-19 might need support for a wide range of complications (7) and evidence shows that follow-up programs and rehabilitation services should be developed to address the impact of “*long haulers*” (8).

Studies published on long COVID-19 until now showed a high degree of heterogeneity. In particular they considered very different follow-up lengths, mainly from 3 to 6 months, discordant inclusion criteria with many cohorts involving both hospitalized and non-hospitalized patients, diverse signs and symptoms investigated and a different approach in patient assessment ranging from hospital visit, dedicated mobile phone applications and telephone interviews to collect self-reported symptoms (4–6, 8–18).

Here, we present a prospective study designed to assess a large class of patient-reported symptoms and sequelae in a homogenous cohort of hospitalized patients after 12 months of follow up.

## Methods

### Study Design and Participants

This is a prospective, observational and multicenter cohort study supported by Fondazione IRCSS Ca' Granda Ospedale Maggiore Policlinico di Milano and Istituto di Ricerche Farmacologiche Mario Negri IRCCS including eight hospitals located in North and Central Italy.

All consecutive adults with a positive RT-PCR result for SARS-CoV-2 admitted to eight hospitals located in North and Central Italy were enrolled in the COVID-19 Network cohort. Patients aged <18 years and pregnant women were excluded.

Data collected included age, sex, comorbidities, signs and symptoms of COVID-19 at admission, hospitalization length, ICU admission, acute complications during hospital stay, and destination after discharge. Acute complications during hospitalization included Acute Respiratory Distress Syndrome (ARDS), infections, Acute Kidney Injury (AKI), cardiologic events, metabolic, hepatic/gastrointestinal, haematologic or neurologic disorders, thrombosis, or acute ischemic-hemorrhagic events. A severity scale was used to stratify patients considering the oxygen support needed: grade 0 (oxygen support not required), grade 1 (oxygen through Venturi mask), and grade 2 [oxygen through High Flow Nasal Cannula (HFNC), CPAP or non-invasive ventilation].

### Outcomes

The follow up was proposed by telephone interview to all COVID-19 Network patients who were discharged alive between the beginning of the pandemic (last week of February 2020) and the 31st May 2020.

Four outcomes were assessed: (i) significant medical problems defined as need of hospitalization or emergency room (ER) access and cardio-neuro-vascular events after the discharge; (ii) patients' subjective perception of general health; (iii) symptoms (ageusia, anosmia, cough, dyspnea at rest, episodes of anxiety, exertional dyspnea (measured by the modified British Medical Research Council (mMRC) dyspnea scale (19), fatigue, gastrointestinal symptoms, headache, loss of appetite, myalgia, limitations on daily life activities, memory problems, sleep disorders); (iv) presence of a group of sequelae which include dyspnea at rest and exertional dyspnea, myalgia, and limitations on daily life activities that we arbitrarily grouped as “*disabling sequelae*” considering their impact on general health status and daily life.

Patient's subjective assessments was recorded on a 0–10 scale to evaluate the generic health status and capacity to appreciate smells and taste (0 = very poor, 10 = very good).

For the remaining symptoms and signs a 0–10 scale was employed where 0 = absence of the symptom and 10 = maximum intensity. A subjective score ≥5 was considered as presence of symptom.

For example, we asked patients direct question like: “*how would you score your state of health from 0 to 10 where 0 is a very bad state of health and 10 is a perfect state of health?*”

The same type of question was asked about the other signs or symptoms: *how would you score your loss of smells from 0 to 10 where 0* = *absence of the symptom and 10*=*maximum subjective intensity?*

Outcomes were assessed at 12 months after discharge from COVID-19 hospitalization. Participants were asked to report current symptoms, i.e., those present in the previous 14 days, except for gastrointestinal symptoms whose presence had to be reported during the 12 months period (from discharge to administration of the questionnaire).

Patients who died during the 12 months from hospital discharge were identified through Regional Health Care System informatic tool.

Questionnaires were administered *via* telephone interviews run by a trained medical investigator. If the subject was unreachable at first call, three attempts were made.

Answers to the questionnaire were filled into the REDCap data capture tool.

The study protocol was approved by the institutional review board (Ethics Committee of Milano Area 2, number 556) and informed consent was provided by all study participants. All procedures were in accordance with the 1964 Helsinki Declaration and its later amendments or comparable ethical standards.

### Statistics

The study population was described with means and standard deviations for continuous variables, and with frequencies and percentages for categorical variables. Characteristics of patients with and without interview were compared using Pearson's chi-squared for categorical variables and *t*-test for the continuous ones.

Logistic regressions were fitted to find which baseline and COVID-19 characteristic were associated with presence of the following outcomes at 12 months: health status deterioration, severe medical problems, and high frequency of sequelae. We first calculated different models corrected for age, sex, number of comorbidities, and ethnicity. Subsequently all significant (*p* < 0.05) variables were included in corrected models.

Statistical analyses were performed using Stata v.15.1 (Stata Corp, College Station, TX). In this study, we followed the strengthening the Reporting of observational studies in epidemiology (STROBE) guidelines (see Supplementary Material).

## Results

**Figure 1:**
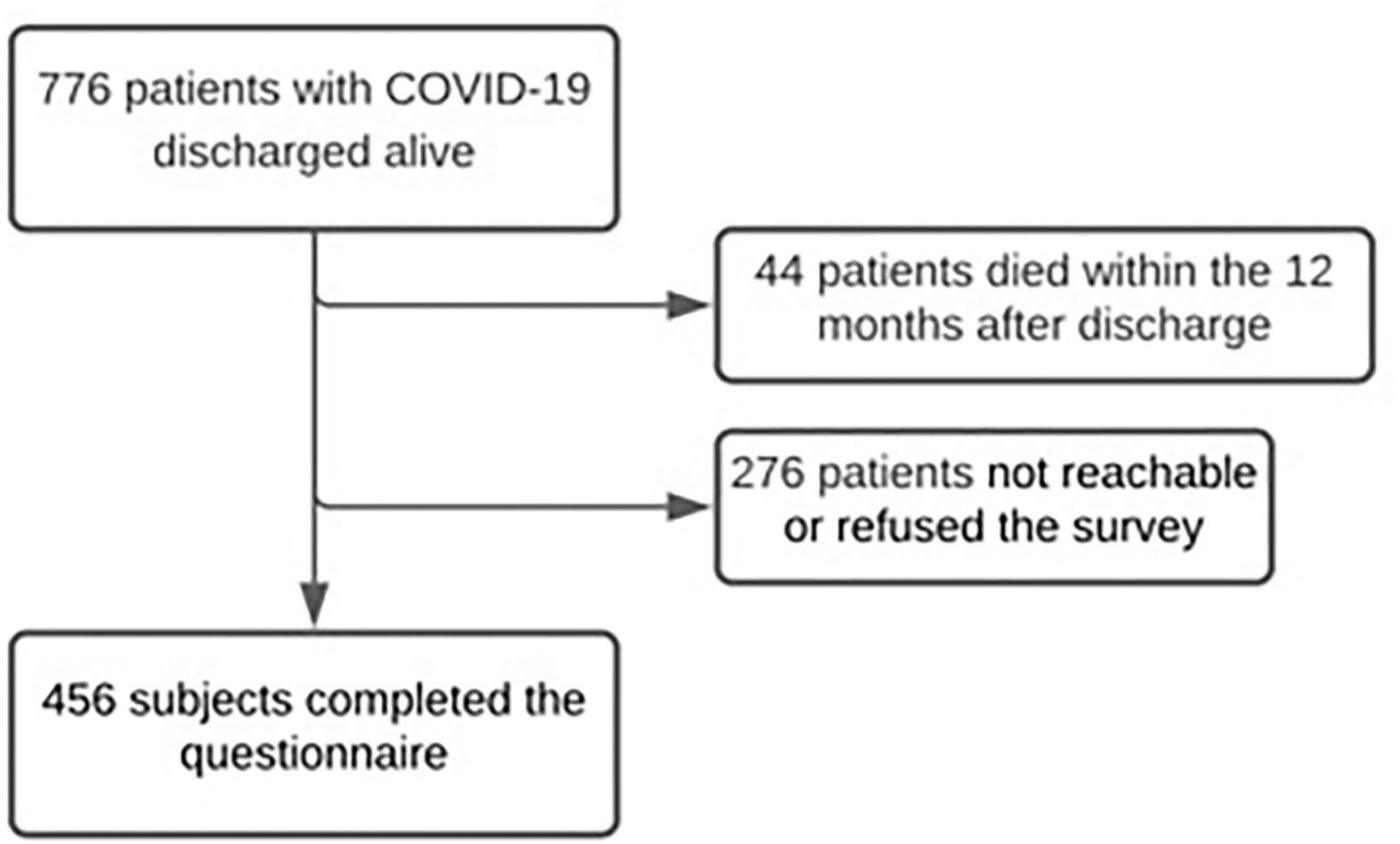
Flow chart of patients with COVID-19 discharged between the pandemic beginning and May 31st, 2020.

A total of 776 patients admitted to hospital because of COVID-19 and discharged alive from February 28 to May 31, 2020 were included in the analysis. Forty-four subjects (5.7%) died during the 12 months after discharge and 276 (35.6%) subjects were unreachable or refused to join the telephone interview. Four hundred and fifty-six individuals completed the questionnaire (58.8%; Figure 1).

Table 1 summarizes the demographic and clinical characteristics of the study population. Patients were mainly of Caucasian origin (87.7%) and male (63.2%). Mean age was 59.4 years (SD 14.1).

**Table 1:** Characteristics of patients hospitalized with COVID-19 and discharged alive who consented to follow up interview.

	**Value**
**Total**, ***N***	456
**Female**, ***N*** **(%)**	168 (36.8%)
**Age, mean (SD)**	59.4 (14.1)
**Age, range (** ***N*** **, %)**	
18–44	62 (13.6%)
45–64	225 (49.3%)
≥65	169 (37.1%)
**Ethnicity**, ***N*** **(%)**	
Caucasian	377 (87.7%)
Other	53 (12.3%)
**Comorbidities**, ***N*** **(%)**	
Respiratory diseases	52 (11.7%)
Cardiovascular diseases	188 (42.2%)
Nephropathies	16 (3.4%)
GI diseases and hepatopathies	37 (8.3%)
Rheumatological diseases	10 (2.3%)
Metabolic diseases	81 (18.2%)
Neurologic diseases	20 (4.5%)
Cancer	16 (3.6%)
SOT and HSCT	5 (1.1%)
**Number of comorbidities**, ***N*** **(%)**	
0	176 (40.4%)
1–2	197 (45.2%)
≥3	63 (14.5%)
**Symptoms at COVID-19 onset**, ***N*** **(%)**	
Respiratory symptoms	364 (80.4%)
Systemic symptoms*	412 (90.4%)
Neurologic symptoms	64 (14.4%)
GI symptoms	85 (18.8%)
**Number of symptoms at COVID-19 onset, median (IQR)**	3 (2–4)
**Hospitalization length, median (IQR)**	12 (6–21)
**Hospitalization length**, ***N*** **(%)**	
<14 days	242 (53.1%)
≥14 days	214 (46.9%)
**ICU admission**, ***N*** **(%)**	46 (10.4%)
**Destination after discharge**, ***N*** **(%)**	
Home	378 (84.4%)
Rehab facility/Long-term care	70 (15.6%)
**Complications during hospital stay**, ***N*** **(%)**	250 (55.0%)
**Severity scale**, ***N*** **(%)**	
1 (H, no oxygen required)	136 (30.2%)
2 (H, O_2_ max Venturi Mask)	224 (49.7%)
3 (H, HFNC or CPAP or NIV)	91 (20.2%)

Nearly 60% of patients had at least one comorbidity with the 14.4% suffering from more than three comorbidities. Cardiovascular diseases were the most common health problem present (42.2%).

The median length of hospital stay was 12.0 (IQR 6.0–21.0) days and 46 subjects (10.4%) were admitted to the intensive care unit (ICU).

One hundred and thirty-six (30.2%) participants did not require oxygen support during their hospital stay, 224 (49.7%) required it through nasal cannula or Venturi mask and 91 (20.2%) needed high-flow oxygen support through HFNC, CPAP or non-invasive ventilation (Table 1).

We compared participants who joined the follow-up to those who were unreachable or refused to join the survey. In the latter group mean age was significantly higher (64 years vs. 59 among the participants, *p* < 0.001). Patients who were unreachable or refused telephone interview were more frequently discharged to rehabilitation centers/nursing homes (*p* < 0.001), had a higher number of comorbidities (*p* = 0.074) and, specifically, had more neurologic (*p* = 0.039) or metabolic (*p* = 0.018) comorbidities, and suffered more from acute complications during the hospitalization in COVID-19 unit (*p* = 0.044) (Supplementary Table S1).

### Patient-Reported Symptoms and Sequelae at 12 Months

Overall, 91.7% of patients reported at least one persisting symptom/sequela 12 months after hospital discharge and 69.6% reported two or more symptoms.

**Figure 2:**
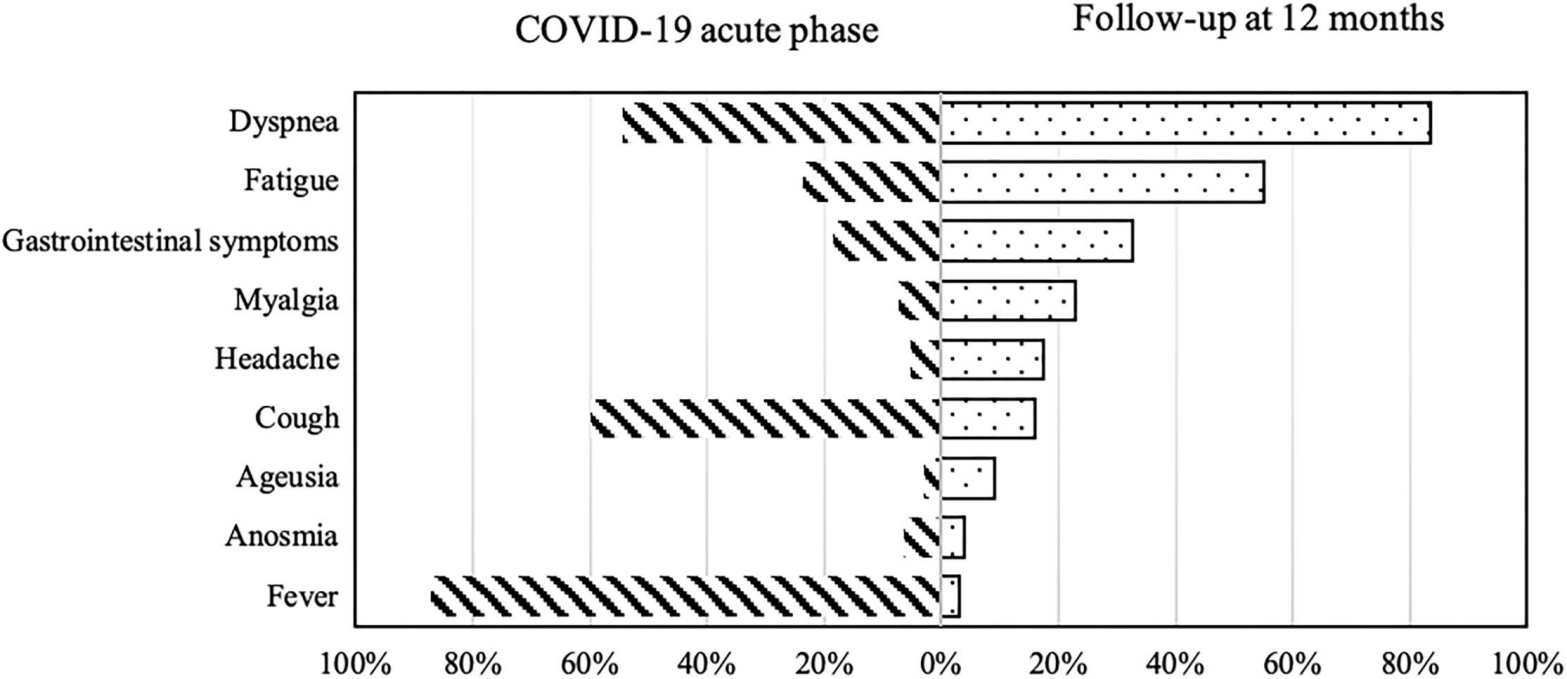
COVID-19-related symptoms during the acute phase of illness (on the left) and at 12-months follow-up (on the right).

Respiratory problems were the most reported symptoms/sequelae with 71.7% of participants suffering from exertional dyspnea (score ≥ 1 in mMRC scale), while 12.5% of participants reported dyspnea at rest (Figure 2).

Other most common symptoms reported at 12 months were fatigue (54.6%), gastrointestinal symptoms (altered bowel habits and bloating) (32.8%), sleep disorders (32.4%), anxiety (23.2%), and myalgias (22.3%; Table 2).

**Table 2:** Persistent symptoms and sequelae investigated among patients with COVID-19, discharged alive and who agreed to follow up interview (*n* = 456).

**General health status after COVID-19, *N* (%)**	**Value**
<5	11 (2.42%)
5–6	65 (14.32%)
7–8	234 (51.54%)
9–10	144 (31.72%)
**Severe medical issues after COVID-19**, ***N*** **(%)**	88 (19.38%)
ER admission	47 (10.35%)
Hospitalization	30 (6.61%)
- Respiratory problems	5 (1.10%)
- Neurologic problems	1 (0.22%)
- Psychiatric problem	0
- GI problems	1 (0.22%)
- Hepatologic problems	2 (0.44%)
- Cardiovascular problems	17 (3.72%)
- Other	15 (3.30%)
Other	15 (3.30%)
**Respiratory symptoms**, ***N*** **(%)**
Dyspnea at rest (≥5)	57 (12.5%)
Exertional dyspnea	
- mMRC 0	128 (28.32%)
- mMRC ≥ 1	324 (71.68%)
Cough	73 (16.08%)
**GI symptoms**, ***N*** **(%)**
Altered gastrointestinal function (altered bowel habits and bloating)	149 (32.75%)
**Other symptoms**, ***N*** **(%)**
Smell disorder (<5)	18 (3.96%)
Taste disorder (<5)	13 (2.86%)
Memory disorder (<5)	15 (3.47%)
Fatigue	230 (54.63%)
Headache	73 (17.38%)
Sleep difficulties	147 (32.38%)
Decreased appetite	34 (7.49%)
Limitations to daily activities (limitations to daily activities + troubled walking)	69 (16.35%)
Anxiety (≥5)	104 (23.16%)
Myalgia	94 (22.27%)

**Figure 3:**
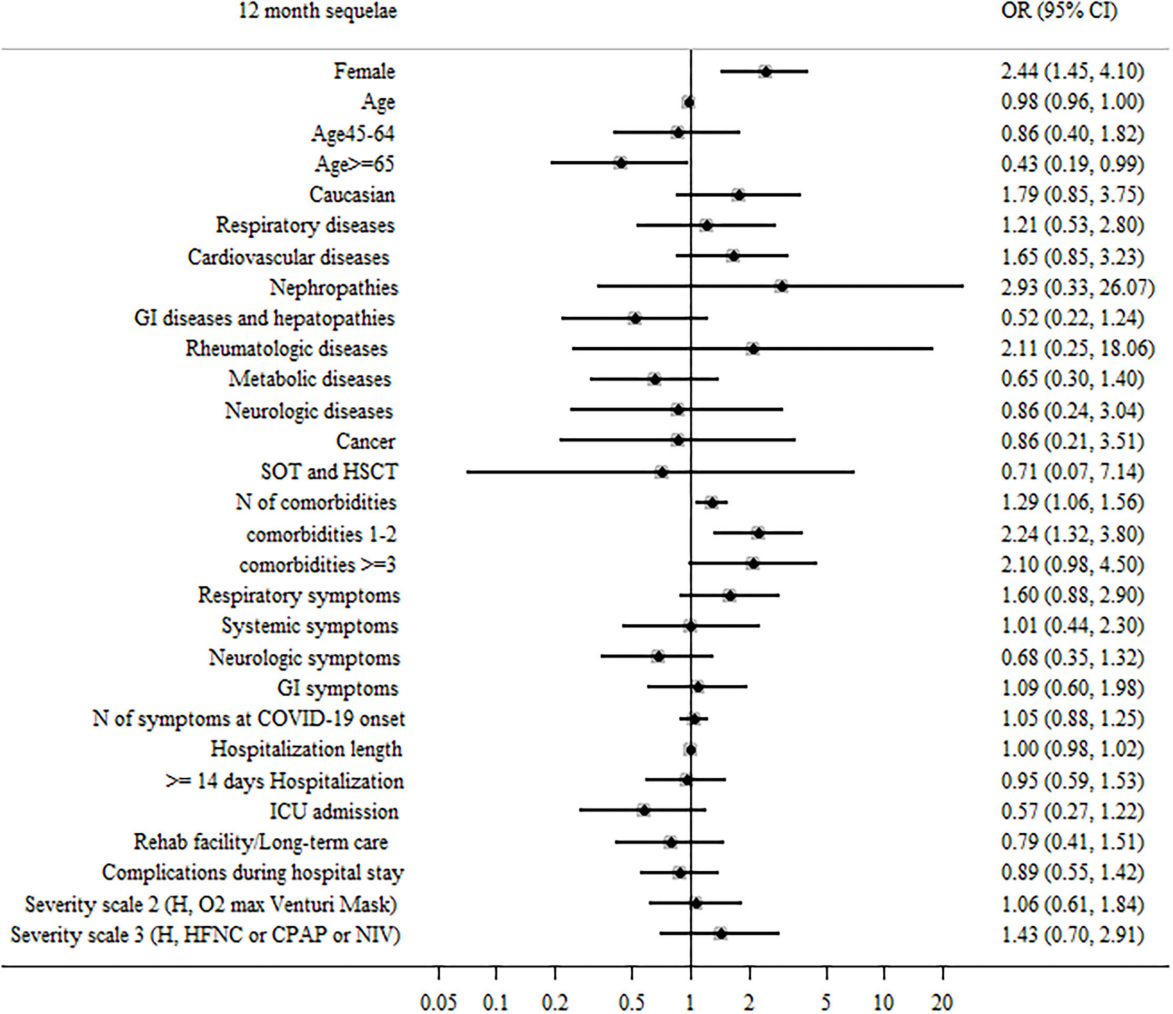
Patient factors associated with ≥2 sequelae or persistent symptoms during the 12 months after hospital discharge in surviving patients.

When comparing participants with 0–1 persisting symptoms at 12 months to those who reported ≥2 symptoms, predictors of ≥2 symptoms reported were: female sex (aOR = 2.44, 95% CI: 1.45–4.1, and *p* = 0.001) and 1–2 comorbidities (aOR = 2.24, 95% CI: 1.32–3.8, and *p* = 0.009), while older age (≥65 years old) showed a protective role (aOR = 0.43, 95% CI: 0.19–0.99, and *p* < 0.027; Figure 3).

When all significant (*p* < 0.05) variables were included in a general multivariable model, female sex, older age, and the presence of 1–2 comorbidities at baseline were confirmed as independent predictors of ≥2 symptoms (Supplementary Table S2).

About 81% patients reported at least one disabling sequelae (dyspnea at rest, exertional dyspnea, myalgia, and limitations on daily life activities) and 30.1% reported two or more.

Female sex (aOR = 2.62, 95% CI: 1.64–4.17, and *p* < 0.001), 1–2 comorbidities at baseline (aOR = 1.19, 95% CI: 1.12–3.27, and *p* = 0.048), number of symptoms in the acute phase (aOR = 1.2, 95% CI: 1.02–1.41, and *p* = 0.031), presence of respiratory symptoms (aOR = 2.05, 95% CI: 1.07–3.93, and *p* = 0.031) and neurologic symptoms (aOR = 1.98, 95% CI: 1.04–3.76, and *p* = 0.038) during COVID-19 hospitalization were associated to the presence of two or more disabling sequelae (Supplementary Table S3).

### General Health Status at 12 Months

**Figure 4:**
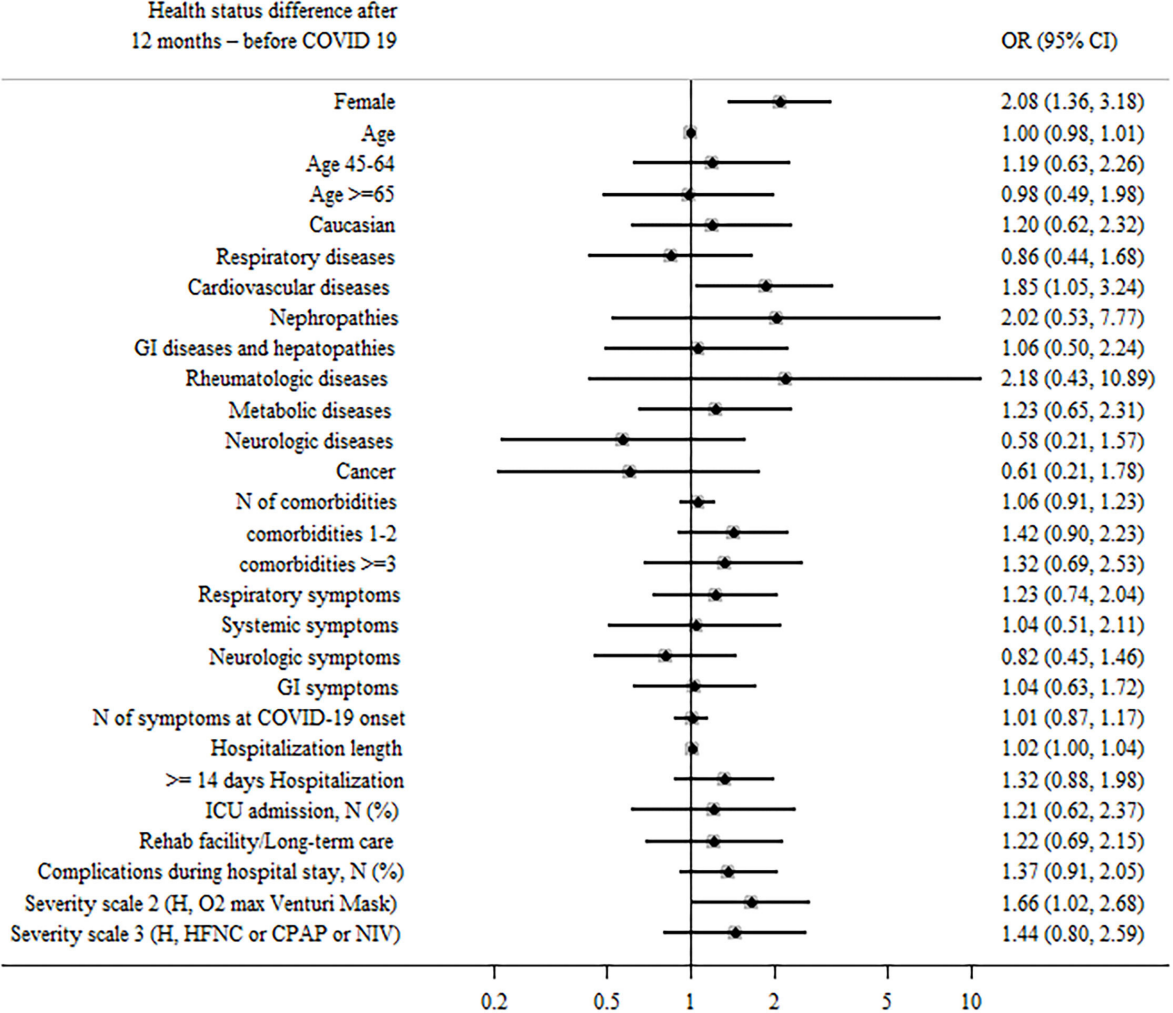
Patient factors associated with health status difference after 12 months—before COVID-19.

Self-perception of general health 12 months after COVID-19 discharge was poor (a score < 5) in 2.4% of patients whereas 31.7% reported an excellent health status (a score ≥9) (Figure 4).

When comparing post- to pre-COVID19 scores of self-perception of general health, 55.5% of participants reported a lower score after COVID-19, while 44.5% described a stable or improved score (38.3% stable health status, 6.2% an improved health condition).

Predictors significantly associated to health impairment were female sex (aOR = 2.08, 95% CI: 1.36–3.18, and *p* = 0.001), cardiovascular comorbidities (aOR = 1.85, 95% CI: 1.05–3.24, and *p* = 0.032), and a longer length of hospital stay (Figure 4).

When all significant (*p* < 0.05) variables were included in a general multivariable model, female sex, and hospitalization length remained as independent predictors of health impairment (Supplementary Table S4).

### Significant Medical Problems After Hospital Discharge

Overall, 19.4% of participants accessed to health care resources during the 12 months after discharge, including: access to ER (10.6% of patients) and need of hospitalization (6.6% of patients; Table 2).

A longer hospital stay during COVID-19 was associated to a higher incidence of significant medical problem after discharge (adjusted aOR = 1.02, 95% CI: 1.003–1.05, and *p* = 0.023) (Supplementary Table S5). No other characteristic was related to presence of significant problem during 12 months after hospital discharge.

## Discussion

In the present study, we showed that ~90% of individuals admitted to hospital for COVID-19 still presents self-reported symptoms 12 months after discharge being respiratory symptoms and fatigue the most common reported sequelae.

To our knowledge, this is one of the largest multicentric and prospective studies on long COVID-19 among patients hospitalized with COVID-19 and followed up systematically after 12 months.

In comparison with other studies, we recorded a very high rate of sequelae. Notably, many of these studies included a large proportion of non-hospitalized patients (4, 6) and others excluded patients who required intubation/mechanical ventilation or reported comorbidities at baseline (11).

In our study, the high rate of COVID-19 patient-reported symptoms/sequelae can be also explained by the large number of areas covered by the survey: beyond respiratory symptoms and fatigue, patients frequently reported systemic symptoms, sleep disorders, and psychological distress.

Moreover, the approach based on subjects' perception we used in this study (telephone interview) and the psychological effects of the subsequent and concurrent COVID-19 waves could overestimate the presence of persisting symptoms.

As confirmed by other authors, female sex, and comorbidities were strongly related to long-COVID19 syndrome (4–6, 9, 12, 14, 17, 20, 21).

In order to explain the correlation between female sex and long COVID-19, physiological and social factors have been reported as possible drivers (15, 22). Nevertheless, Wu et al. (11) demonstrated that female sex strongly predicts impaired DLCO (diffusion lung capacity for carbon monoxide) at 12 months after discharge, suggesting that a physio-pathological process might be present. Moreover, Seeßle et al. proposes a potential role of autoimmunity disorders as co-factor in long COVID incidence and, if proven, it could partially explain the female predisposition to such a syndrome.

Interestingly, and in sharp contrast with other studies (6, 9, 23), age seems to have a protective role on self-perception of symptoms/sequelae after 12 months. A UK study on long COVID-19 supported this finding, indeed they reported that the prevalence of self-reported sequelae was greatest in people aged 35–69 years (24). It is possible that younger patients dedicate more attention to possible signs of health impairment (22), they are probably more reliable in the telephone interview and, at present, we cannot exclude that the development of long COVID could be secondary to an altered immune response possibly more pronounced in younger individuals.

In our study, more than 50% of patients referred health impairment after COVID-19. Interestingly, those subjects frequently present cardiovascular comorbidities. Therefore, the pre-existence of a cardiovascular disease might be a predictive factor of a harder rehabilitation probably because of the effect of SARS-CoV-2 pneumonia on the cardiovascular system (25).

Interestingly, our findings show that the severity of acute COVID-19 (ICU stay, severity scale, and complications during hospitalization) does not have a significant impact on long COVID-19.

Thus far, available findings on long COVID-19 incidence do not differ so much between non-hospitalized subjects and hospitalized ones (6, 9, 17, 18). This data, shared by several groups, deserves attention and could be an interesting starting point for further analysis.

As experienced in many other contexts during the present pandemic, most studies dealing with post-COVID-19 have employed surveys by telephone call or mobile Apps (5, 6, 9, 16–18, 26, 27).

In our study, nearly 50% refused the survey or were not reachable but, unfortunately, we do not know the correct proportion of the two sub-population. Those people were fragile patients, older, and/or hosted in long-term rehabilitation centers.

In some studies, subjects with expected communication impairment were excluded (12, 26) allowing authors to be more selective and, consequently, the percentage of reported COVID-19 sequelae resulted lower. In conclusion, our findings are probably more representative of a younger and heathier population while fragile subjects are underrepresented in our study and in all similar ones. Targeted studies are needed.

Our study has several limitations. First, the absence of a control group and of an intermediate follow up time point makes it difficult to highlight improving or worsening trend.

Second, by focusing on hospitalized patients, symptoms reported after 12 months could be attributable to either COVID-19 or by the hospitalization, making it difficult to identify the direct consequence of COVID-19. Third, telephone interviews did not permit any objective measures of patient symptoms or signs but, conversely, self-reported symptoms could represent a selection tool to identify those patients who may benefit from an outpatient assessment.

By contrast, some strengths are evident. This is one of the few available prospective studies on COVID-19 follow-up which evaluated health status of a large and homogenous cohort of previously hospitalized patients at 12 months after discharge in Lombardy, one of the most affected regions in Europe during the first wave.

The questionnaire, within the limits of its structure, allowed to investigate many areas of one's health status.

Moreover, all data were collected prospectively, both during acute COVID-19 and during follow up in order to avoid possible bias during retrospective collection of hospitalization documents.

To conclude, in this study we highlighted the existence of a long COVID-19 pandemic lasting 12 months after hospitalization and emphasize the need to implement public-health strategies to manage this condition. Future studies are likely to clarify the chronic nature of long-COVID-19 and, consequently, the need for large-scale economic investments in terms of public health and social policies.

## Data Availability Statement

The raw data supporting the conclusions of this article will be made available by the authors, without undue reservation.

## Ethics Statement

The studies involving human participants were reviewed and approved by Ethics Committee of Milano Area 2, number 556. The patients/participants provided their written informed consent to participate in this study.

## COVID-19 Network” Working Group

Fondazione IRCCS Ca' Granda Ospedale Maggiore Policlinico. Silvano Bosari, Luigia Scudeller, Giuliana Fusetti, Laura Rusconi, and Silvia Dell'Orto: Scientific Direction. Daniele Prati, Luca Valenti, Silvia Giovannelli, Maria Manunta, Giuseppe Lamorte, and Francesca Ferarri: Department of Transfusion Medicine and Hematology (Biobank). Andrea Gori, Alessandra Bandera, Antonio Muscatello, Davide Mangioni, Laura Alagna, Giorgio Bozzi, Andrea Lombardi, Riccardo Ungaro, Giuseppe Ancona, Marco Mussa, Bianca Veronica Mariani, Matteo Bolis, Nathalie Iannotti, Serena Ludovisi, Agnese Comelli, Giulia Renisi, Simona Biscarini, Valeria Castelli, Emanuele Palomba, Marco Fava, Valeria Fortina, Carlo Alberto Peri, Paola Saltini, Giulia Viero, Teresa Itri, Valentina Ferroni, Valeria Pastore, Roberta Massafra, Arianna Liparoti, Toussaint Muheberimana, Alessandro Giommi, Rosaria Bianco, Rafaela Montalvao De Azevedo, and Grazia Eliana Chitani: Infectious Diseases Unit. Flora Peyvandi, Roberta Gualtierotti, Barbara Ferrari, Raffaella Rossio, Nadia Boasi, Erica Pagliaro, Costanza Massimo, Michele De Caro, and Andrea Giachi: Angelo Bianchi Bonomi Hemophilia and Thrombosis Center and Fondazione Luigi Villa. Nicola Montano, Barbara Vigone, Chiara Bellocchi, Angelica Carandina, Elisa Fiorelli, Valerie Melli, and Eleonora Tobaldini: UOC Internal Medicine, Immunology and Allergology. Francesco Blasi, Stefano Aliberti, Maura Spotti, Leonardo Terranova, Sofia Misuraca, Alice D'Adda, Silvia Della Fiore, Marta Di Pasquale, Marco Mantero, Martina Contarini, Margherita Ori, Letizia Morlacchi, Valeria Rossetti, Andrea Gramegna, Maria Pappalettera, Mirta Cavallini, and Agata Buscemi: Respiratory Unit and Cystic Fibrosis Adult Center. Marco Vicenzi, Irena Rota, Giorgio Costantino, Monica Solbiati, Ludovico Furlan, Marta Mancarella, Giulia Colombo, Giorgio Colombo, Alice Fanin, and Mariele Passarella: Cardiology Unit. Valter Monzani, Ciro Canetta, Angelo Rovellini, Laura Barbetta, Filippo Billi, Christian Folli, and Silvia Accordino: Acute Internal Medicine. Diletta Maira, Cinzia Maria Hu, Irene Motta, and Natalia Scaramellini: Rare Diseases Center. Anna Ludovica Fracanzani, Rosa Lombardi, and Annalisa Cespiati: General Medicine and Metabolic Diseases. Matteo Cesari, Tiziano Lucchi, Marco Proietti, Laura Calcaterra, Clara Mandelli, Carlotta Coppola, and Arturo Cerizza: Geriatric Unit. Antonio Maria Pesenti, Giacomo Grasselli, and Alessandro Galazzi: Intensive Care Unit. Elisa Colombo, Filippo Cantù, Valentina Tombola, and Francesca Gallo: Psychiatry Unit. Alessandro Nobili, Mauro Tettamanti, Igor Monti, and Alessia Antonella Galbussera: Istituto di Ricerche Farmacologiche Mario Negri IRCCS. Ernesto Crisafulli, Domenico Girelli, Alessio Maroccia, Daniele Gabbiani, Fabiana Busti, Alice Vianello, Marta Biondan, and Filippo Sartori: Policlinico G.B. Rossi, Verona: UOC di Medicina d'Urgenza. Paola Faverio, Alberto Pesci, and Stefano Zucchetti: Ospedale San Gerardo, ASST Monza: UOC Pneumologia. Paolo Bonfanti, Marianna Rossi, Ilaria Beretta, and Anna Spolti: Malattie Infettive. Sergio Harari: San Giuseppe Hospital MultiMedica IRCCS and community health, Università degli Studi di Milano: UOC Pneumologia. Davide Elia: Unità di Pneumologia e terapia Semi-intensiva respiratoria, Servizio di Fisiopatologia Respiratoria ed Emodinamica Polmonare. Roberto Cassandro and Antonella Caminati: Unità di Pneumologia e terapia Semi-intensiva respiratoria, Servizio di Fisiopatologia Respiratoria ed Emodinamica Polmonare. Francesco Cipollone, Maria Teresa Guagnano, Damiano D'Ardes, Ilaria Rossi, and Francesca Vezzani: Ospedale Clinicizzato “SS. Annunziata”: Clinica Medica. Antonio Spanevello, Francesca Cherubino, and Dina Visca: ICS Maugeri Tradate, Universita' Insubria: Pneumologia Riabilitativa. Marco Contoli, Alberto Papi, Luca Morandi, and Nicholas Battistini: Azienda Ospedaliera Universitaria di Ferrara e Dipartimento di Medicina Traslazionale Università di Ferrara: UO Pneumologia. Guido Luigi Moreo and Pasqualina Iannuzzi: Clinica Polispecialistica San Carlo: UO Medicina Interna. Daniele Fumagalli: UO Oncologia. Sara Leone: UO Chirurgia Generale.

## Author Contributions

AC: conceptualization, data curation, and original draft writing. GV: original draft writing and data curation. GB: data curation and investigation. AN: conceptualization and methodology. MT: conceptualization, methodology, and formal analysis. AGa: methodology and formal analysis. AM: conceptualization and supervision. MM, CC, PBr, PBo, and MC: review, editing, and investigation. AGo: validation and supervision. AB: conceptualization, validation, and supervision. All authors contributed to the article and approved the submitted version.

## Conflict of Interest

The authors declare that the research was conducted in the absence of any commercial or financial relationships that could be construed as a potential conflict of interest.

## Publisher's Note

All claims expressed in this article are solely those of the authors and do not necessarily represent those of their affiliated organizations, or those of the publisher, the editors and the reviewers. Any product that may be evaluated in this article, or claim that may be made by its manufacturer, is not guaranteed or endorsed by the publisher.

## References

[1] Cortinovis M Perico N Remuzzi G. Long-term follow-up of recovered patients with COVID-19. Lancet. (2021) 397:173–5. 10.1016/S0140-6736(21)00039-8 33428868 PMC7833833

[2] World Health Organization. A Clinical Case Definition of Post COVID- 19 Condition by a Delphi Consensus. Geneva: World Health Organization (2021). Available online at: https://www.who.int/publications/i/item/WHO-2019-nCoV-Post_COVID-19_condition-Clinical_case_definition-2021.1 (accessed November 25, 2021).

[3] The Lancet. Facing up to long COVID. Lancet. (2020) 396:1861. 10.1016/S0140-6736(20)32662-3 33308453 PMC7834723

[4] Seeßle J Waterboer T Hippchen T Simon J Kirchner M Lim A. Persistent symptoms in adult patients 1 year after coronavirus disease 2019 (COVID-19): a prospective cohort study. Clin Infect Dis. (2021) ciab611. 10.1093/cid/ciab611 34223884 PMC8394862

[5] Xiong Q Xu M Li J Liu Y Zhang J Xu Y. Clinical sequelae of COVID-19 survivors in Wuhan, China: a single-centre longitudinal study. Clin Microbiol Infect. (2021) 27:89–95. 10.1016/j.cmi.2020.09.023 32979574 PMC7510771

[6] Boscolo-Rizzo P Guida F Polesel J Marcuzzo AV Capriotti V D'Alessandro A. Sequelae in adults at 12 months after mild-to-moderate coronavirus disease 2019 (COVID-19). Int Forum Allergy Rhinol. (2021) 11:1685–8. 10.1002/alr.22832 34109765 PMC9291310

[7] Tansey CM. One-year outcomes and health care utilization in survivors of severe acute respiratory syndrome. Arch Intern Med. (2007) 167:1312. 10.1001/archinte.167.12.1312 17592106

[8] Halpin SJ McIvor C Whyatt G Adams A Harvey O McLean L. Postdischarge symptoms and rehabilitation needs in survivors of COVID-19 infection: a cross-sectional evaluation. J Med Virol. (2021) 93:1013–22. 10.1002/jmv.26368 32729939

[9] Sudre CH Murray B Varsavsky T Graham MS Penfold RS Bowyer RC. Attributes and predictors of long COVID. Nat Med. (2021) 27:626–31. 10.1038/s41591-021-01292-y 33692530 PMC7611399

[10] Taquet M Geddes JR Husain M Luciano S Harrison PJ. 6-month neurological and psychiatric outcomes in 236 379 survivors of COVID-19: a retrospective cohort study using electronic health records. Lancet Psychiatry. (2021) 8:416–27. 10.1016/S2215-0366(21)00084-5 33836148 PMC8023694

[11] Wu X Liu X Zhou Y Yu H Li R Zhan Q. 3-month, 6-month, 9-month, and 12-month respiratory outcomes in patients following COVID-19-related hospitalisation: a prospective study. Lancet Respir Med. (2021) 9:747–54. 10.1016/S2213-2600(21)00174-0 33964245 PMC8099316

[12] Huang C Huang L Wang Y Li X Ren L Gu X. 6-month consequences of COVID-19 in patients discharged from hospital: a cohort study. Lancet. (2021) 397:220–32. 10.1016/S0140-6736(20)32656-8 33428867 PMC7833295

[13] Carfì A Bernabei R Landi F. Persistent symptoms in patients after acute COVID-19. JAMA. (2020) 324:603. 10.1001/jama.2020.12603 32644129 PMC7349096

[14] Ghosn J Piroth L Epaulard O Le Turnier P Mentré F Bachelet D. Persistent COVID-19 symptoms are highly prevalent 6 months after hospitalization: results from a large prospective cohort. Clin Microbiol Infect. (2021) 27:1041.e1–e4. 10.1016/j.cmi.2021.03.012 34125067 PMC8107834

[15] Goërtz YMJ Van Herck M Delbressine JM Vaes AW Meys R Machado FVC. Persistent symptoms 3 months after a SARS-CoV-2 infection: the post-COVID-19 syndrome? ERJ Open Res. (2020) 6:00542–2020. 10.1183/23120541.00542-2020 33257910 PMC7491255

[16] Logue JK Franko NM McCulloch DJ McDonald D Magedson A Wolf CR. Sequelae in adults at 6 months after COVID-19 infection. JAMA Netw Open. (2021) 4:e210830. 10.1001/jamanetworkopen.2021.0830 33606031 PMC7896197

[17] Peghin M Palese A Venturini M De Martino M Gerussi V Graziano E. Post-COVID-19 symptoms 6 months after acute infection among hospitalized and non-hospitalized patients. Clin Microbiol Infect. (2021) 27:1507–13. 10.1016/j.cmi.2021.05.033 34111579 PMC8180450

[18] Righi E Mirandola M Mazzaferri F Razzaboni E Zaffagnini A Erbogasto A. Long-term patient-centred follow-up in a prospective cohort of patients with COVID-19. Infect Dis Ther. (2021) 10:1579–90. 10.1007/s40121-021-00461-3 34152573 PMC8215633

[19] Mahler DA Wells CK. Evaluation of clinical methods for rating dyspnea. Chest. (1988) 93:580–6. 10.1378/chest.93.3.580 3342669

[20] Sykes DL Holdsworth L Jawad N Gunasekera P Morice AH Crooks MG. Post-COVID-19 symptom burden: what is long-COVID and how should we manage it? Lung. (2021) 199:113–9. 10.1007/s00408-021-00423-z 33569660 PMC7875681

[21] Bai F Tomasoni D Falcinella C Barbanotti D Castoldi R Mulè G. Female gender is associated with long COVID syndrome: a prospective cohort study. Clin Microbiol Infect. (2021) 293. 10.1016/j.cmi.2021.11.002 34763058 PMC8575536

[22] Bardel A Wallander M-A Wallman T Rosengren A Johansson S Eriksson H. Age and sex related self-reported symptoms in a general population across 30 years: patterns of reporting and secular trend. PLoS One. (2019) 14:e0211532. 10.1371/journal.pone.0211532 30716129 PMC6361431

[23] Huang C Wang Y Li X Ren L Zhao J Hu Y. Clinical features of patients infected with 2019 novel coronavirus in Wuhan, China. Lancet. (2020) 395:497–506. 10.1016/S0140-6736(20)30183-5 31986264 PMC7159299

[24] Ayoubkhani D Bosworth M. Prevalence of Ongoing Symptoms Following Coronavirus (COVID-19) infectiOn in the UK. Office for National Statistics Bullettin (2021). p. 1–16. Available online at: https://www.ons.gov.uk/peoplepopulationandcommunity/healthandsocialcare/conditionsanddiseases/bulletins/prevalenceofongoingsymptomsfollowingcoronaviruscovid19infectionintheuk/4november2021 (accessed December 10, 2021) (cited November 21, 2021).

[25] Puntmann VO Carerj ML Wieters I Fahim M Arendt C Hoffmann J. Outcomes of cardiovascular magnetic resonance imaging in patients recently recovered from coronavirus disease 2019 (COVID-19). JAMA Cardiol. (2020) 5:1265. 10.1001/jamacardio.2020.3557 32730619 PMC7385689

[26] Weerahandi H Hochman KA Simon E Blaum C Chodosh J Duan E. Post-discharge health status and symptoms in patients with severe COVID-19. J Gen Intern Med. (2021) 36:738–45. 10.1007/s11606-020-06338-4 33443703 PMC7808113

[27] Romero-Duarte Á Rivera-Izquierdo M Guerrero-Fernández de Alba I Pérez-Contreras M Fernández-Martínez NF Ruiz-Montero R. Sequelae, persistent symptomatology and outcomes after COVID-19 hospitalization: the ANCOHVID multicentre 6-month follow-up study. BMC Med. (2021) 19:129. 10.1186/s12916-021-02003-7 34011359 PMC8134820

