# Supplementary material for: Patient-Reported Symptoms and Sequelae 12 Months After COVID-19 in Hospitalized Adults: A Multicenter Long-Term Follow-Up Study
Source: Front Med (Lausanne). 2022 Mar 22;9:834354. doi: 10.3389/fmed.2022.834354 (PMC8981315; doi:10.3389/fmed.2022.834354)
Supplement: Supplementary file 1 [file Table_1.DOCX]

Supplementary Material

**Supplementary Table 1** Comparison between patients hospitalized with COVID-19 and discharged alive who consented to follow up interview and who refused/were not reachable

|  | **Refused/not reachable** | **12 months follow-up available** | **p-value** |
| --- | --- | --- | --- |
| **Total, N** | 276 | 456 |  |
| **Female, N (%)** | 92 (33.3%) | 168 (36.8%) | 0.336 |
| **Age, mean (SD)** | 63.8 (16.3) | 59.4 (14.1) | 0.0001 |
| **Age, range (N, %)** |  |  |  |
| 18-44 | 30 (10.9%) | 62 (13.6%) | 0.012 |
| 45-64 | 113 (40.9%) | 225 (49.3%) |  |
| >=65 | 133 (48.2%) | 169 (37.1%) |  |
| **Ethnicity, N (%)** |  |  | 0.271 |
| Caucasian | 222 (84.7%) | 377 (87.7%) |  |
| Other | 40 (15.3%) | 53 (12.3%) |  |
| **Comorbidities, N (%)** |  |  |  |
| Respiratory diseases | 27 (10.1%) | 52 (11.7%) | 0.524 |
| Cardiovascular diseases | 126 (47.2%) | 188 (42.2%) | 0.19 |
| Nephropathies | 18 (6.8%) | 16 (3.4%) | 0.054 |
| GI diseases and hepatopathies | 20 (7.6%) | 37 (8.3%) | 0.744 |
| Rheumatological diseases | 4 (1.5%) | 10 (2.3%) | 0.49 |
| Metabolic diseases | 68 (25.7%) | 81 (18.2%) | 0.018 |
| Neurologic diseases | 22 (8.2%) | 20 (4.5%) | 0.039 |
| Cancer | 11 (4.2%) | 16 (3.6%) | 0.704 |
| SOT and HSCT | 2 (0.8%) | 5 (1.1%) | 0.626 |
| **Number of comorbidities, N (%)** |  |  | 0.074 |
| 0 | 98 (39.5%) | 176 (40.4%) |  |
| 1-2 | 98 (39.5%) | 197 (45.2%) |  |
| >=3 | 52 (21.0%) | 63 (14.5%) |  |
| **Symptoms at COVID-19 onset, N (%)** |  |  |  |
| Respiratory symptoms | 208 (75.6%) | 364 (80.4%) | 0.133 |
| Systemic symptoms* | 246 (89.1%) | 412 (90.4%) | 0.596 |
| Neurologic symptoms | 33 (12.5%) | 64 (14.4%) | 0.481 |
| GI symptoms | 55 (20.2%) | 85 (18.8%) | 0.641 |
| **Number of symptoms at COVID-19 onset, median (IQR)** | 3 (2-3) | 3 (2-4) | 0.165 |
| **Hospitalization length, median (IQR)** | 14 (8-22) | 12 (6-21) | 0.239 |
| **Hospitalization length, N (%)** |  |  |  |
| <14 days | 137 (49.6%) | 242 (53.1%) | 0.368 |
| >= 14 days | 139 (50.4%) | 214 (46.9%) |  |
| **ICU admission, N (%)** | 37 (14.0%) | 46 (10.4%) | 0.15 |
| **Destination after discharge, N (%)** |  |  | <0.001 |
| Home | 189 (70.5%) | 378 (84.4%) |  |
| Rehab facility/Long-term care | 79 (29.5%) | 70 (15.6%) |  |
| **Complications during hospital stay, N (%)** | 172 (62.6%) | 250 (55.0%) | 0.044 |
| **Severity scale, N (%)** |  |  | 0.602 |
| 1 (H, no oxygen required) | 89 (32.4%) | 136 (30.2%) |  |
| 2 (H, O2 max Venturi Mask) | 126 (45.8%) | 224 (49.7%) |  |
| 3 (H, HFNC or CPAP or NIV) | 60 (21.8%) | 91 (20.2%) |  |
| *=fever, myalgia, fatigue, arthralgia | | | |
