# Supplementary material for: Patient-Reported Symptoms and Sequelae 12 Months After COVID-19 in Hospitalized Adults: A Multicenter Long-Term Follow-Up Study
Source: Front Med (Lausanne). 2022 Mar 22;9:834354. doi: 10.3389/fmed.2022.834354 (PMC8981315; doi:10.3389/fmed.2022.834354)
Supplement: Supplementary file 2 [file Table_2.docx]

Supplementary Material

Supplementary Table 2 ﻿Patient factors associated with ≥2 sequelae or persistent symptoms during the 12 months after hospital discharge in surviving patients

|  | 0-1 | >=2 | unadjusted  OR (95% C.I.) | unadjusted  p-value | Age, sex, comorbidity and Caucasian adjusted  OR (95% C.I.) | Age, sex, comorbidity and Caucasian adjusted  p-value | Fully adjusted (all variables with p<0.005) |
| --- | --- | --- | --- | --- | --- | --- | --- |
| **Total, N** | 124 | 284 | <=408 |  | <=368 |  |  |
| **Female, N (%)** | 30 (24.2%) | 125 (44.0%) | 2.46 (1.53-3.95) | <0.001 | 2.44 (1.45-4.10) | 0.001 | 2.44 (1.49-4.00) |
| **Age, Mean (SD)** | 59.5 (15.0) | 58.4 (13.5) | 0.99 (0.98-1.01) | 0.457 | 0.98 (0.96-0.998) | 0.034 | 0.98 (0.97-1.002) |
| **Age, N (%)** |  |  |  |  |  |  |  |
| 18-44 | 17 (13.7%) | 42 (14.8%) | ref | 0.457 |  | 0.027 |  |
| 45-64 | 58 (46.8%) | 148 (52.1%) | 1.03 (0.54-1.96) |  | 0.86 (0.4-1.82) |  |  |
| >=65 | 49 (39.5%) | 94 (33.1%) | 0.78 (0.40-1.50) |  | 0.43 (0.19-0.99) |  |  |
| **Ethnicity, N (%)** |  |  |  | 0.910 |  | 0.124 |  |
| Caucasian | 97 (86.6%) | 235 (87.0%) | 1.04 (0.54-1.99) |  | 1.79 (0.85-3.75) |  |  |
| Other | 15 (13.4%) | 35 (13.0%) | ref |  |  |  |  |
| **Comorbidities, N (%)** |  |  |  |  |  |  |  |
| Respiratory diseases | 10 (8.3%) | 40 (14.4%) | 1.87 (0.90-3.87) | 0.093 | 1.21 (0.53-2.80) | 0.650 |  |
| Cardiovascular diseases | 40 (33.1%) | 121 (43.5%) | 1.56 (1-2.44) | 0.051 | 1.65 (0.85-3.23) | 0.140 |  |
| Nephropathies | 1 (0.8%) | 13 (4.7%) | 5.89 (0.76-45.52) | 0.089 | 2.93 (0.33-26.07) | 0.335 |  |
| GI diseases and hepatopathies | 11 (9.1%) | 21 (7.6%) | 0.82 (0.38-1.75) | 0.604 | 0.52 (0.22-1.24) | 0.141 |  |
| Rheumatologic diseases | 1 (0.8%) | 8 (2.9%) | 3.56 (0.44-28.74) | 0.234 | 2.11 (0.25-18.06) | 0.497 |  |
| Metabolic diseases | 21 (17.4%) | 51 (18.4%) | 1.07 (0.61-1.88) | 0.801 | 0.65 (0.30-1.40) | 0.273 |  |
| Neurologic diseases | 4 (3.3%) | 12 (4.3%) | 1.32 (0.42-4.18) | 0.637 | 0.86 (0.24-3.04) | 0.816 |  |
| Cancer | 3 (2.5%) | 9 (3.2%) | 1.32 (0.35-4.95) | 0.684 | 0.86 (0.21-3.51) | 0.836 |  |
| SOT and HSCT | 1 (0.8%) | 4 (1.4%) | 1.75 (0.19-15.84) |  | 0.71 (0.07-7.14) |  |  |
| **N of comorbidities, N (%)** |  |  |  | 0.018 |  | 0.009 |  |
| 0 | 61 (51.7%) | 100 (36.8%) | ref |  |  |  |  |
| 1-2 | 41 (34.8%) | 133 (48.9%) | 1.98 (1.23-3.18) |  | 2.24 (1.32-3.80) |  | 2.35 (1.41-3.91) |
| >=3 | 16 (13.6%) | 39 (14.3%) | 1.49 (0.77-2.89) |  | 2.10 (0.98-4.50) |  | 2.04 (0.97-4.27) |
| **Symptoms at COVID-19 onset, N (%)** |  |  |  |  |  |  |  |
| Respiratory symptoms | 93 (78.9%) | 236 (83.1%) | 1.48 (0.88-2.50) | 0.143 | 1.60 (0.88-2.90) | 0.125 |  |
| Systemic symptoms | 112 (90.3%) | 253 (89.1%) | 0.87 (0.43-1.77) | 0.708 | 1.01 (0.44-2.30) | 0.979 |  |
| Neurologic symptoms | 18 (15.3%) | 39 (13.9%) | 0.90 (0.49-1.65) | 0.730 | 0.68 (0.35-1.32) | 0.251 |  |
| GI symptoms | 20 (16.4%) | 57 (20.1%) | 1.28 (0.73-2.24) | 0.387 | 1.09 (0.60-1.98) | 0.780 |  |
| **N of symptoms at COVID-19 onset, median (IQR)** | 3 (2-4) | 3 (2-4) | 1.06 (0.91-1.24) | 0.449 | 1.05 (0.88-1.25) | 0.603 |  |
| **Hospitalization length, median (IQR)** | 12 (8-21) | 11 (5-20) | 0.996 (0.98-1.01) | 0.658 | 1.002 (0.98-1.02) | 0.828 |  |
| **Hospitalization length, N (%)** |  |  |  |  |  |  |  |
| <14 days | 66 (53.2%) | 164 (57.8%) | ref | 0.397 |  | 0.839 |  |
| >= 14 days | 58 (46.8%) | 120 (42.3%) | 0.83 (0.54-1.27) |  | 0.95 (0.59-1.53) |  |  |
| **ICU admission, N (%)** | 14 (11.7%) | 25 (9.1%) | 0.76 (0.38-1.51) | 0.431 | 0.57 (0.27-1.22) | 0.147 |  |
| **Destination after discharge, N (%)** |  |  |  | 0.841 |  | 0.476 |  |
| Home | 104 (84.6%) | 232 (83.8%) | ref |  |  |  |  |
| Rehab facility/Long-term care | 19 (15.5%) | 45 (16.3%) | 1.06 (0.59-1.90) |  | 0.79 (0.41-1.51) |  |  |
| **Complications during hospital stay, N (%)** | 70 (56.5%) | 151 (53.4%) | 0.88 (0.58-1.35) | 0.564 | 0.89 (0.55-1.42) | 0.619 |  |
| **Severity scale, N (%)** |  |  |  | 0.304 |  | 0.575 |  |
| 1 (H, no oxygen required) | 42 (34.4%) | 86 (30.5%) | ref |  |  |  |  |
| 2 (H, O2 max Venturi Mask) | 62 (50.8%) | 136 (48.2%) | 1.07 (0.67-1.72) |  | 1.06 (0.61-1.84) |  |  |
| 3 (H, HFNC or CPAP or NIV) | 18 (14.8%) | 60 (21.3%) | 1.63 (0.86-3.10) |  | 1.43 (0.70-2.91) |  |  |
