# Supplementary material for: Patient-Reported Symptoms and Sequelae 12 Months After COVID-19 in Hospitalized Adults: A Multicenter Long-Term Follow-Up Study
Source: Front Med (Lausanne). 2022 Mar 22;9:834354. doi: 10.3389/fmed.2022.834354 (PMC8981315; doi:10.3389/fmed.2022.834354)
Supplement: Supplementary file 3 [file Table_3.DOCX]

Supplementary Material

Supplementary Table 3 Frequency of disabling sequelae during the 12 months after hospital discharge in surviving patients

|  | 0-1 | 2+ | unadjusted  OR (95% C.I.) | Unadjusted  p-value | Age, sex, comorbidity and Caucasian adjusted  OR (95% C.I.) | Age, sex, comorbidity and Caucasian adjusted  p-value | Fully adjusted (all variables with p<0.005) |
| --- | --- | --- | --- | --- | --- | --- | --- |
| **Total, N** | 293 | 126 | <=419 |  | <=378 |  |  |
| **Female, N (%)** | 84 (28.7%) | 72 (57.1%) | 3.32 (2.15-5.12) | <0.001 | 2.62 (1.64-4.17) | <0.001 | 2.77 (1.72-4.48) |
| **Age – Mean (SD)** | 58.6 (13.9) | 59.9 (14.2) | 1.01 (0.99-1.02) | 0.389 | 1.01 (0.99-1.02) | 0.565 |  |
| **Age, N (%)** |  |  |  |  |  |  |  |
| 18-44 | 40 (13.7%) | 19 (15.1%) | ref | 0.508 |  | 0.874 |  |
| 45-64 | 153 (52.2%) | 58 (46.0%) | 0.80 (0.43-1.49) |  | 0.90 (0.44-1.84) |  |  |
| >=65 | 100 (34.1%) | 49 (38.9%) | 1.03 (0.54-1.96) |  | 1.02 (0.46-2.24) |  |  |
| **Ethnicity, N (%)** |  |  |  | 0.002 |  | 0.029 |  |
| Caucasian | 248 (90.5%) | 94 (79.0%) | 0.39 (0.22-0.72) |  | 0.46 (0.23-0.92) |  | 0.52 (0.26-1.01) |
| Other | 26 (9.5%) | 25 (21.0%) | ref |  |  |  |  |
| **Comorbidities, N (%)** |  |  |  |  |  |  |  |
| Respiratory diseases | 28 (9.8%) | 23 (18.9%) | 2.15 (1.18-3.91) | 0.012 | 1.65 (0.81-3.35) | 0.164 |  |
| Cardiovascular diseases | 114 (39.7%) | 53 (43.4%) | 1.17 (0.76-1.79) | 0.484 | 1.08 (0.56-2.06) | 0.825 |  |
| Nephropathies | 8 (2.8%) | 6 (4.9%) | 1.8 (0.61-5.31) | 0.285 | 1.02 (0.27-3.88) | 0.980 |  |
| GI diseases and hepatopathies | 19 (6.6%) | 13 (10.7%) | 1.68 (0.80-3.53) | 0.168 | 1.26 (0.55-2.88) | 0.582 |  |
| Rheumatological diseases | 6 (2.1%) | 3 (2.5%) | 1.18 (0.29-4.8) | 0.816 | 0.57 (0.11-3.03) | 0.512 |  |
| Metabolic diseases | 53 (18.5%) | 20 (16.4%) | 0.86 (0.49-1.52) | 0.606 | 0.47 (0.22-1.01) | 0.052 |  |
| Neurologic diseases | 9 (3.1%) | 7 (5.7%) | 1.88 (0.68-5.17) | 0.221 | 1.42 (0.47-4.32) | 0.539 |  |
| Cancer | 9 (3.1%) | 4 (3.3%) | 1.05 (0.32-3.47) | 0.940 | 0.96 (0.26-3.52) | 0.953 |  |
| SOT and HSCT | 3 (1.1%) | 2 (1.6%) | 1.58 (0.26-9.56) |  | 1.10 (0.16-7.42) |  |  |
| **N of comorbidities, N (%)** |  |  |  | 0.061 |  | 0.048 |  |
| 0 | 127 (45.0%) | 38 (32.2%) | ref |  |  |  |  |
| 1-2 | 118 (41.8%) | 61 (51.7%) | 1.73 (1.07-2.78) |  | 1.91 (1.12-3.27) |  | 1.96 (1.16-3.33) |
| >=3 | 37 (13.1%) | 19 (16.1%) | 1.72 (0.89-3.32) |  | 1.98 (0.94-4.21) |  | 2.19 (1.07-4.47) |
| **Symptoms at COVID-19 onset, N (%)** |  |  |  |  |  |  |  |
| Respiratory symptoms | 229 (79.0%) | 107 (84.9%) | 1.5 (0.85-2.64) | 0.159 | 2.05 (1.07-3.93) | 0.031 | 1.76 (0.92-3.36) |
| Systemic symptoms | 264 (90.1%) | 112 (88.9%) | 0.88 (0.45-1.73) | 0.708 | 1.23 (0.56-2.69) | 0.611 |  |
| Neurologic symptoms | 34 (12.0%) | 25 (19.8%) | 1.81 (1.03-3.19) | 0.039 | 1.98 (1.04-3.76) | 0.038 | 2.01 (1.04-3.88) |
| GI symptoms | 52 (17.9%) | 26 (20.6%) | 1.20 (0.71-2.02) | 0.506 | 1.06 (0.60-1.89) | 0.837 |  |
| **N of symptoms at COVID-19 onset, median (IQR)** | 3 (2-4) | 3 (2-4) | 1.12 (0.97-1.30) | 0.123 | 1.2 (1.02-1.41) | 0.031 |  |
| **Hospitalization length, median (IQR)** | 12 (6-21) | 13 (6-19) | 0.999 (0.98-1.02) | 0.935 | 1.004 (0.98-1.03) | 0.682 |  |
| **Hospitalization length, N (%)** |  |  |  |  |  |  |  |
| <14 days | 163 (55.6%) | 70 (55.6%) | ref | 0.989 |  | 0.736 |  |
| >= 14 days | 130 (44.4%) | 56 (44.4%) | 1.003 (0.66-1.53) |  | 1.08 (0.68-1.74) |  |  |
| **ICU admission, N (%)** | 28 (9.8%) | 12 (10.0%) | 1.02 (0.50-2.09) | 0.948 | 1.01 (0.44-2.31) | 0.981 |  |
| **Destination after discharge, N (%)** |  |  |  | 0.510 |  | 0.594 |  |
| Home | 244 (84.7%) | 101 (82.1%) | ref |  |  |  |  |
| Rehab facility/Long-term care | 44 (15.3%) | 22 (17.9%) | 1.21 (0.69-2.12) |  | 0.84 (0.44-1.60) |  |  |
| **Complications during hospital stay, N (%)** | 166 (56.9%) | 63 (50.0%) | 0.76 (0.50-1.15) | 0.197 | 0.72 (0.45-1.16) | 0.180 |  |
| **Severity scale, N (%)** |  |  |  | 0.763 |  | 0.756 |  |
| 1 (H, no oxygen required) | 92 (31.8%) | 39 (31.0%) | ref |  |  |  |  |
| 2 (H, O2 max Venturi Mask) | 144 (49.8%) | 60 (47.6%) | 0.98 (0.61-1.59) |  | 1.10 (0.63-1.90) |  |  |
| 3 (H, HFNC or CPAP or NIV) | 53 (18.3%) | 27 (21.4%) | 1.20 (0.66-2.18) |  | 1.29 (0.66-2.53) |  |  |
