# Supplementary material for: Patient-Reported Symptoms and Sequelae 12 Months After COVID-19 in Hospitalized Adults: A Multicenter Long-Term Follow-Up Study
Source: Front Med (Lausanne). 2022 Mar 22;9:834354. doi: 10.3389/fmed.2022.834354 (PMC8981315; doi:10.3389/fmed.2022.834354)
Supplement: Supplementary file 4 [file Table_4.docx]

Supplementary Material

**Supplementary Table 4** ﻿Patient factors associated with health status difference after 12 months – before COVID 19

|  | <0^a^ | ≥ 0^b^ | unadjusted  OR (95% C.I.) | unadjusted  p-value | Age, sex, comorbidity and Caucasian adjusted  OR (95% C.I.) | Age, sex, comorbidity and Caucasian adjusted  p-value | Fully adjusted (all variables with p<0.005) |  |
| --- | --- | --- | --- | --- | --- | --- | --- | --- |
| **Total, N** | 252 | 202 | <=454 |  | <=413 |  |  |  |
| **Female, N (%)** | 112 (44.4%) | 55 (27.2%) | 2.11 (1.42-3.14) | <0.001 | 2.08 (1.36-3.18) | 0.001 | 2.21 (1.48-3.30) |  |
| **Age – Mean (SD)** | 59.2 (13.5) | 59.7 (15.0) | 0.997 (0.98-1.01) | 0.660 | 0.996 (0.98-1.01) | 0.646 |  |  |
| **Age, range (N, %)** |  |  |  |  |  |  |  |  |
| 18-44 | 33 (13.1%) | 29 (14.4%) | Ref | 0.786 |  | 0.659 |  |  |
| 45-64 | 128 (50.8%) | 95 (47.0%) | 1.16 (0.66-2.04) |  | 1.19 (0.63-2.26) |  |  |  |
| >=65 | 91 (36.1%) | 78 (38.6%) | 1.03 (0.57-1.84) |  | 0.98 (0.49-1.98) |  |  |  |
| **Ethnicity, N (%)** |  |  |  | 0.675 |  | 0.586 |  |  |
| Caucasian | 209 (87.1%) | 167 (88.4%) | 0.88 (0.49-1.58) |  | 1.20 (0.62-2.32) |  |  |  |
| Other | 31 (12.9%) | 22 (11.6%) | Ref |  |  |  |  |  |
| **Comorbidities, N (%)** |  |  |  |  |  |  |  |  |
| Respiratory diseases | 29 (11.7%) | 23 (11.8%) | 0.997 (0.56-1.79) | 0.993 | 0.86 (0.44-1.68) | 0.652 |  |  |
| Cardiovascular diseases | 112 (45.0%) | 76 (39.0%) | 1.30 (0.89-1.90) | 0.174 | 1.85 (1.05-3.24) | 0.032 |  |  |
| Nephropathies | 10 (4.0%) | 6 (3.1%) | 1.33 (0.48-3.73) | 0.585 | 2.02 (0.53-7.77) | 0.306 |  |  |
| GI diseases and hepatopathies | 23 (9.2%) | 14 (7.2%) | 1.33 (0.67-2.66) | 0.419 | 1.06 (0.50-2.24) | 0.888 |  |  |
| Rheumatological diseases | 8 (3.2%) | 2 (1.0%) | 3.22 (0.68-15.34) | 0.142 | 2.18 (0.43-10.89) | 0.344 |  |  |
| Metabolic diseases | 48 (19.3%) | 33 (17.0%) | 1.18 (0.72-1.92) | 0.508 | 1.23 (0.65-2.31) | 0.522 |  |  |
| Neurologic diseases | 9 (3.6%) | 11 (5.6%) | 0.63 (0.26-1.56) | 0.322 | 0.58 (0.21-1.57) | 0.281 |  |  |
| Cancer | 8 (3.2%) | 8 (4.1%) | 0.78 (0.29-2.13) | 0.633 | 0.61 (0.21-1.78) | 0.364 |  |  |
| SOT and HSCT | 5 (2.0%) | 0 | - |  |  |  |  |  |
| **N of comorbidities, N (%)** |  |  |  | 0.284 |  | 0.311 |  |  |
| 0 | 92 (37.4%) | 82 (43.6%) | Ref |  |  |  |  |  |
| 1-2 | 119 (48.4%) | 78 (41.5%) | 1.39 (0.92-2.10) |  | 1.42 (0.90-2.23) |  |  |  |
| >=3 | 35 (14.2%) | 28 (14.9%) | 1.14 (0.64-2.03) |  | 1.32 (0.69-2.53) |  |  |  |
| **Symptoms at COVID-19 onset, N (%)** |  |  |  |  |  |  |  |  |
| Respiratory symptoms | 206 (81.8%) | 156 (78.4%) | 1.22 (0.77-1.94) | 0.404 | 1.23 (0.74-2.04) | 0.429 |  |  |
| Systemic symptoms | 227 (90.1%) | 183 (90.6%) | 0.93 (0.50-1.75) | 0.827 | 1.04 (0.51-2.11) | 0.915 |  |  |
| Neurologic symptoms | 35 (14.2%) | 29 (14.7%) | 0.97 (0.57-1.66) | 0.918 | 0.82 (0.45-1.46) | 0.495 |  |  |
| GI symptoms | 51 (20.4%) | 33 (16.4%) | 1.27 (0.78-2.05) | 0.335 | 1.04 (0.63-1.72) | 0.885 |  |  |
| **N of symptoms at COVID-19 onset, median (IQR)** | 3 (2-4) | 3 (2-4) | 1.03 (0.91-1.18) | 0.614 | 1.01 (0.87-1.17) | 0.897 |  |  |
| **Hospitalization length, median (IQR)** | 13 (6.5-22) | 12 (6-20) | 1.01 (0.996-1.03) | 0.149 | 1.02 (1.00001-1.04) | 0.050 | 1.02 (0.999-1.03) |  |
| **Hospitalization length, N (%)** |  |  |  |  |  |  |  |  |
| <14 days | 130 (51.6%) | 112 (55.5%) | Ref | 0.481 |  | 0.182 |  |  |
| >= 14 days | 122 (48.4%) | 90 (44.6%) | 1.14 (0.79-1.65) |  | 1.32 (0.88-1.98) |  |  |  |
| **ICU admission, N (%)** | 28 (11.6%) | 17 (8.5%) | 1.32 (0.71-2.47) | 0.380 | 1.21 (0.62-2.37) | 0.577 |  |  |
| **Destination after discharge, N (%)** |  |  |  | 0.171 |  | 0.497 |  |  |
| Home | 204 (82.3%) | 172 (86.9%) | ref |  |  |  |  |  |
| Rehab facility/Long-term care | 44 (17.7%) | 26 (13.1%) | 1.44 (0.85-2.44) |  | 1.22 (0.69-2.15) |  |  |  |
| **Complications during hospital stay, N (%)** | 143 (56.8%) | 105 (52.2%) | 1.18 (0.81-1.71) | 0.390 | 1.37 (0.91-2.05) | 0.130 |  |  |
| **Severity scale, N (%)** |  |  |  | 0.154 |  | 0.121 |  |  |
| 1 (H, no oxygen) | 66 (26.4%) | 69 (34.7%) | ref |  |  |  |  |  |
| 2 (H, O2 max Venturi Mask) | 131 (52.4%) | 92 (46.2%) | 1.49 (0.97-2.29) |  | 1.66 (1.02-2.68) |  |  |  |
| 3 (H, HFNC or CPAP or NIV) | 53 (21.2%) | 38 (19.1%) | 1.48 (0.87-2.53) |  | 1.44 (0.8-2.59) |  |  |  |
| ^a^ <0: the post COVID-19 score is deteriorated  ^b^ >=0: the post COVID-19 score is stable, ameliorated | | | | | | | |  |
