# Supplementary material for: Patient-Reported Symptoms and Sequelae 12 Months After COVID-19 in Hospitalized Adults: A Multicenter Long-Term Follow-Up Study
Source: Front Med (Lausanne). 2022 Mar 22;9:834354. doi: 10.3389/fmed.2022.834354 (PMC8981315; doi:10.3389/fmed.2022.834354)
Supplement: Supplementary file 5 [file Table_5.docx]

Supplementary Material

Supplementary Table 5 Severe medical problems during the 12 months of follow up after hospital discharge

|  | yes | no | Unadjusted  OR (95% C.I.) | Unadjusted  p-value | Age, sex, comorbidity and Caucasian adjusted  OR (95% C.I.) | Age, sex, comorbidity and Caucasian adjusted  p-value | Fully adjusted (all variables with p<0.005) |
| --- | --- | --- | --- | --- | --- | --- | --- |
| **Total, N** | 88 | 366 | <=454 |  | <=412 |  |  |
| **Female, N (%)** | 39 (44.7%) | 128 (35.0%) | 1.48 (0.92-2.37) | 0.104 | 1.36 (0.81-2.29) | 0.241 |  |
| **Age, Mean (SD)** | 60.0 (13.1) | 59.2 (14.4) | 1.004 (0.99-1.02) | 0.639 | 0.99 (0.97-1.02) | 0.628 |  |
| **Age - range, N (%)** |  |  |  |  |  |  |  |
| 18-44 | 9 (10.2%) | 53 (14.5%) | ref | 0.445 |  | 0.139 |  |
| 45-64 | 48 (54.6%) | 176 (48.1%) | 1.61 (0.74-3.49) |  | 1.61 (0.68-3.80) |  |  |
| >=65 | 31 (35.2%) | 137 (37.4%) | 1.33 (0.59-2.99) |  | 0.93 (0.36-2.43) |  |  |
| **Ethnicity, N (%)** |  |  |  | 0.753 |  | 0.792 |  |
| Caucasian | 71 (86.6%) | 304 (87.9%) | 0.89 (0.44-1.82) |  | 1.12 (0.49-2.56) |  |  |
| Other | 11 (13.4%) | 42 (12.1%) | ref |  |  |  |  |
| **Comorbidities, N (%)** |  |  |  |  |  |  |  |
| Respiratory diseases | 10 (11.9%) | 42 (11.7%) | 1.02 (0.49-2.13) | 0.951 | 0.89 (0.38-2.06) | 0.784 |  |
| Cardiovascular diseases | 36 (42.9%) | 152 (42.2%) | 1.03 (0.64-1.66) | 0.916 | 0.75 (0.37-1.53) | 0.432 |  |
| Nephropathies | 7 (8.3%) | 9 (2.5%) | 3.55 (1.28-9.81) | 0.015 | 2.63 (0.67-10.29) | 0.165 | 3.41 (1.23-9.49) |
| GI diseases and hepatopathies | 7 (8.3%) | 29 (8.1%) | 1.04 (0.44-2.46) | 0.933 | 0.94 (0.37-2.38) | 0.900 |  |
| Rheumatological diseases | 1 (1.2%) | 9 (2.5%) | 0.48 (0.06-3.81) | 0.484 | - |  |  |
| Metabolic diseases | 16 (19.1%) | 65 (18.1%) | 1.06 (0.58-1.95) | 0.841 | 1.06 (0.49-2.29) | 0.879 |  |
| Neurologic diseases | 2 (2.4%) | 18 (5.0%) | 0.46 (0.11-2.04) | 0.309 | 0.18 (0.02-1.46) | 0.109 |  |
| Cancer | 5 (6.0%) | 11 (3.1%) | 2.01 (0.68-5.94) | 0.208 | 2.19 (0.7-6.86) | 0.180 |  |
| SOT and HSCT | 2 (2.4%) | 3 (0.8%) | 2.90 (0.48-17.65) |  | 2.50 (0.38-16.62) |  |  |
| **N of comorbidities, N (%)** |  |  |  | 0.389 |  | 0.359 |  |
| 0 | 27 (34.2%) | 148 (41.7%) | ref |  |  |  |  |
| 1-2 | 41 (51.9%) | 155 (43.7%) | 1.45 (0.85-2.48) |  | 1.52 (0.85-2.72) |  |  |
| >=3 | 11 (13.9%) | 52 (14.7%) | 1.16 (0.54-2.50) |  | 1.29 (0.56-3.02) |  |  |
| **Symptoms at COVID-19 onset, N (%)** |  |  |  |  |  |  |  |
| Respiratory symptoms | 67 (77.9%) | 295 (80.8%) | 0.84 (0.47-1.48) | 0.542 | 0.80 (0.43-1.48) | 0.474 |  |
| Systemic symptoms | 77 (87.5%) | 333 (91.0%) | 0.69 (0.34-1.43) | 0.323 | 0.74 (0.33-1.66) | 0.461 |  |
| Neurologic symptoms | 13 (14.9%) | 51 (14.3%) | 1.05 (0.54-2.04) | 0.876 | 1.14 (0.56-2.34) | 0.720 |  |
| GI symptoms | 20 (22.7%) | 64 (17.7%) | 1.37 (0.78-2.41) | 0.277 | 1.36 (0.74-2.48) | 0.320 |  |
| **N of symptoms at COVID-19 onset, median (IQR)** | 3 (2-4) | 3 (2-4) | 0.93 (0.79-1.10) | 0.410 | 0.93 (0.77-1.12) | 0.444 |  |
| **Hospitalization length, median (IQR)** | 14 (8.5-23.5) | 12 (6-20) | 1.02 (1.0001-1.04) | 0.049 | 1.02 (1.003-1.05) | 0.023 | 1.02 (1-1.04) |
| **Hospitalization length, N (%)** |  |  |  |  |  |  |  |
| <14 days | 43 (48.9%) | 199 (54.4%) | ref | 0.353 |  | 0.348 |  |
| >= 14 days | 45 (51.1%) | 167 (45.6%) | 1.25 (0.78-1.99) |  | 1.28 (0.77-2.13) |  |  |
| **ICU admission, N (%)** | 13 (15.1%) | 32 (9.0%) | 1.79 (0.90-3.58) | 0.099 | 1.81 (0.84-3.89) | 0.128 |  |
| **Destination after discharge, N (%)** |  |  |  | 0.244 |  | 0.649 |  |
| Home | 70 (86.5%) | 307 (85.5%) | ref |  |  |  |  |
| Rehab facility/Long-term care | 17 (19.5%) | 52 (14.5%) | 1.43 (0.78-2.63) |  | 1.17 (0.59-2.34) |  |  |
| **Complications during hospital stay, N (%)** | 53 (60.2%) | 196 (53.7%) | 1.31 (0.81-2.10) | 0.270 | 1.23 (0.73-2.06) | 0.439 |  |
| **Severity scale, N (%)** |  |  |  | 0.567 |  | 0.391 |  |
| 1 (H, no oxygen required) | 22 (25.8%) | 114 (31.3%) | ref |  |  |  |  |
| 2 (H, O2 max Venturi Mask) | 46 (54.1%) | 176 (48.4%) | 1.35 (0.77-2.37) |  | 1.55 (0.82-2.94) |  |  |
| 3 (H, HFNC or CPAP or NIV) | 17 (20.0%) | 74 (20.3%) | 1.19 (0.59-2.39) |  | 1.24 (0.57-2.71) |  |  |
